# Supplementary material for: MetaRibo-Seq measures translation in microbiomes
Source: Nat Commun. 2020 Jun 29;11:3268. doi: 10.1038/s41467-020-17081-z (PMC7324362; doi:10.1038/s41467-020-17081-z)
Supplement: Supplementary file 10 — Supplementary Data 7 [file 41467_2020_17081_MOESM10_ESM.zip › File2/Confidence_High_Taxonomy/349684_out.krona.html]

Javascript must be enabled to view this page.

members
magnitude
magnitudeUnassigned
count
unassigned
taxon
rank

349684\_out

9

2
superkingdom
9

32066
phylum
9

9
203490
class

order
203491
9

9
1129771
family

32067
genus
9

9

SRS019219\_contig\_number\_20663SRS022530\_contig\_number\_contig-100\_3486.74789.74789SRS042643\_contig\_number\_contig-100\_5014.132945.132945SRS063288\_contig\_number\_contig-100\_2320.128291SRS098045\_contig\_number\_7831SRS143310\_contig\_number\_51941SRS143354\_contig\_number\_12784SRS144090\_contig\_number\_contig-100\_16795.16796SRS147094\_contig\_number\_22413
species
1785996
